# A Quantitative Tractography Study Into the Connectivity, Segmentation and Laterality of the Human Inferior Longitudinal Fasciculus

**DOI:** 10.3389/fnana.2018.00047

**Published:** 2018-06-05

**Authors:** Sandip S. Panesar, Fang-Cheng Yeh, Timothée Jacquesson, William Hula, Juan C. Fernandez-Miranda

**Affiliations:** ^1^Department of Neurological Surgery, University of Pittsburgh, Pittsburgh, PA, United States; ^2^Department of Bioengineering, University of Pittsburgh, Pittsburgh, PA, United States; ^3^Veterans Affairs Pittsburgh Healthcare System, Pittsburgh, PA, United States

**Keywords:** inferior longitudinal fasciculus, tractography, semantic anatomy, white matter anatomy, non-tensor tractography

## Abstract

The human inferior longitudinal fasciculus (ILF) is a ventral, temporo-occipital association tract. Though described in early neuroanatomical works, its existence was later questioned. Application of *in vivo* tractography to the neuroanatomical study of the ILF has generally confirmed its existence, however, consensus is lacking regarding its subdivision, laterality and connectivity. Further, there is a paucity of detailed neuroanatomic data pertaining to the exact anatomy of the ILF. Generalized Q-Sampling imaging (GQI) is a non-tensor tractographic modality permitting high resolution imaging of white-matter structures. As it is a non-tensor modality, it permits visualization of crossing fibers and accurate delineation of close-proximity fiber-systems. We applied deterministic GQI tractography to data from 30 healthy subjects and a large-volume, averaged diffusion atlas, to delineate ILF anatomy. Post-mortem white matter dissection was also carried out in three cadaveric specimens for further validation. The ILF was found in all 60 hemispheres. At its occipital extremity, ILF fascicles demonstrated a bifurcated, ventral-dorsal morphological termination pattern, which we used to further subdivide the bundle for detailed analysis. These divisions were consistent across the subject set and within the atlas. We applied quantitative techniques to study connectivity strength of the ILF at its anterior and posterior extremities. Overall, both morphological divisions, and the un-separated ILF, demonstrated strong leftward-lateralized connectivity patterns. Leftward-lateralization was also found for ILF volumes across the subject set. Due to connective and volumetric leftward-dominance and ventral location, we postulate the ILFs role in the semantic system. Further, our results are in agreement with functional and lesion-based postulations pertaining to the ILFs role in facial recognition.

## Introduction

The human inferior longitudinal fasciculus (ILF) is a ventral, temporo-occipital association tract. Historically, it was described as connecting the superior, middle, inferior and fusiform gyri, to the lingual, cuneate, lateral-occipital and occipito-polar cortices. Early descriptions came from post-mortem white matter dissection (Dejerine and Dejerine-Klumpke, 1895; Crosby, 1962; Gloor, 1997). More recently, radioisotopic tracer studies in non-human primates questioned the existence of a robust temporo-occipital fasciculus, proposing instead a series of cortico-cortical U-fibers, traveling along the antero-posterior temporal distance (Tusa and Ungerleider, 1985; Catani et al., 2003). The introduction of tractography somewhat reconciled this controversy: Early diffusion tensor imaging (DTI) studies confirmed the existence of the human ILF (Catani et al., 2002, 2003; Mori et al., 2002; Jellison et al., 2004; Kier et al., 2004; Wakana et al., 2004; Fernández-Miranda et al., 2008; Urbanski et al., 2008). Catani et al. (2002, 2003) described a bi-component structure, with a direct temporo-occipital sub-tract, and an indirect series of cortico-cortical U-fibers. The direct subfascicle demonstrated a tri-pronged posterior, and bi-pronged anterior connectivity profile. This connective and structural description remained largely unchanged until a recent dissection and DTI series by Latini (2015) and Latini et al. (2017). The authors proposed a rightward-lateralized ILF arrangement derived from its posterior connectivity profile: dorsally, distinct ILF subfasciculi originated from the cuneate and lateral occipital lobes, while ventrally, subfasciculi originated from the fusiform and lingual gyri (Latini, 2015; Latini et al., 2017).

Prior to the introduction of functional neuroimaging, data pertaining to ILF function was derived primarily from lesional data. Visual agnosia and amnesia were attributed to bilateral damage to ventral temporal white matter. Prosopagnosia (Benson, 1974; Bauer, 1984; Michel et al., 1989; Grossi et al., 2014) and visual hypo-emotionality (Habib, 1986; Takeuchi et al., 2013; Fischer et al., 2016) were linked to right-sided ventral temporal lesions. Further insights have come from both functional neuroimaging (e.g., fMRI) and intraoperative electrical stimulation (IES) (Duffau et al., 2005; Mandonnet et al., 2007). fMRI studies demonstrated semantic activations within temporo-polar, anterior temporal, basal occipito-temporal and occipital lobes corresponding to dominant-hemisphere ILF trajectory (Saur et al., 2008, 2010). An IES study by Mandonnet et al. (2007) proposed the ILF’s role in semantic functionality, consistent with the ‘dorsal-ventral’ model of language organization (Hickok and Poeppel, 2015).

Tractography permits functional insights by studying cortical-connectivity patterns of white matter tracts. It also allows calculation of volumetric, lateralization and subdivision patterns of white matter systems. It therefore surpasses post-mortem dissection in terms of ability and accuracy (Fernandez-Miranda et al., 2012). DTI is unable to delineate crossing fibers and demonstrate cortical connectivity accurately (Fernandez-Miranda et al., 2012; Farquharson et al., 2013). Generalized q-sampling imaging (GQI) dispenses with the diffusion tensor, allowing crossing fibers at close proximity to be tracked at high-resolution (Yeh et al., 2010, 2013). Our group has pioneered the application of GQI tractography to neuroanatomical (Wang et al., 2013, 2016; Fernández-Miranda et al., 2015; Meola et al., 2016; Yoshino et al., 2016; Panesar et al., 2017) and surgical studies (Fernandez-Miranda et al., 2012). Further, our use of large-volume GQI-derived atlases (Yeh and Tseng, 2011; Wang et al., 2013; Fernández-Miranda et al., 2015; Panesar et al., 2017) provides a model of ‘average’ white matter anatomy for validation. With these considerations in mind, we set out to study the subdivision, asymmetry and connectivity of the human ILF using GQI tractography using quantitative connectometry methods, along with diffusion atlas and dissection validation.

## Materials and Methods

### Participants

We conducted a subject-specific deterministic fiber tractography study in 30 right-handed, neurologically healthy male and female subjects, age range 23–35. The data were from the Human Connectome Project (HCP) online database [WU-Minn Consortium (Principal Investigators: David van Essen and Kamil Ugurbil; 1U54MH091657)] funded by the 16 NIH institutes and centers that support the NIH Blueprint for Neuroscience Research and by the McDonnell Center for Systems Neuroscience at Washington University. Likewise, data from 1021 individual HCP subjects were utilized to compile the averaged diffusion atlas.

### Image Acquisition and Reconstruction

The HCP diffusion data for individual subjects were acquired using a Siemens 3T Skyra system, with a 32-channel head coil (Siemans Medical, Erlangen, Germany). A multi-shell diffusion scheme was used, and the *b*-values were 1000, 2000, and 3000 s/mm^2^. The number of diffusion sampling directions were 90, 90, and 90, respectively. The in-plane resolution and slice thickness were both 1.25 mm (TR = 5500 ms, TE = 89 ms, resolution = 1.25 mm × 1.25 mm, FoV = 210 mm × 180 mm, matrix = 144 × 168). The DSI data were reconstructed using the generalized q-sampling imaging approach (Yeh et al., 2010) using a diffusion distance ratio of 1.2 as recommended by the original study.

### HCP 1021 Atlas

A total of 1021 participants from the HCP database were used to construct the atlas. The image acquisition parameters are identical to those described previously. The diffusion data were reconstructed and warped to the Montreal Neurological Institute (MNI) space using *q-*space diffeomorphic reconstruction (Yeh and Tseng, 2011) with a diffusion sampling length ratio of 1.25 and output resolution of 1 mm. The group average atlas was then constructed by averaging the reconstructed data of the 1021 individual subjects within the MNI space.

### Fiber Tracking and Analysis

We performed deterministic fiber tracking using DSI Studio software, which utilizes a generalized streamline fiber tracking method (Yeh et al., 2013). Parameters selected for fiber tracking included a step size of 0.2 mm, a minimum fiber length of 20 mm and a turning angle threshold of 60°. For progression locations containing >1 fiber orientation, fiber orientation most congruent with the incoming direction and turning angle <60° was selected to determine subsequent moving direction. Each progressive voxels’ moving directional estimate was weighted by 20% of the previous voxels incoming direction and by 80% of its nearest fiber orientation. This sequence was repeated to create fiber tracts. Termination of the tracking algorithm occurred when the quantitative anisotropy (QA) (Yeh et al., 2013) dropped below a subject-specific value: when fiber tract continuity no longer met the progression criteria, or when 100,000 tracts were generated. We pre-selected QA termination threshold, between 0.02 and 0.08, by analyzing the number of false continuities generated within each subjects’ dataset and chose the compromise value that allowed optimal anatomical detail with minimal noise. Likewise, we selected a smoothing parameter of 50% for the same reason stated previously.

### Region of Interest Placement and Fiber Selection

**FIGURE 1:**
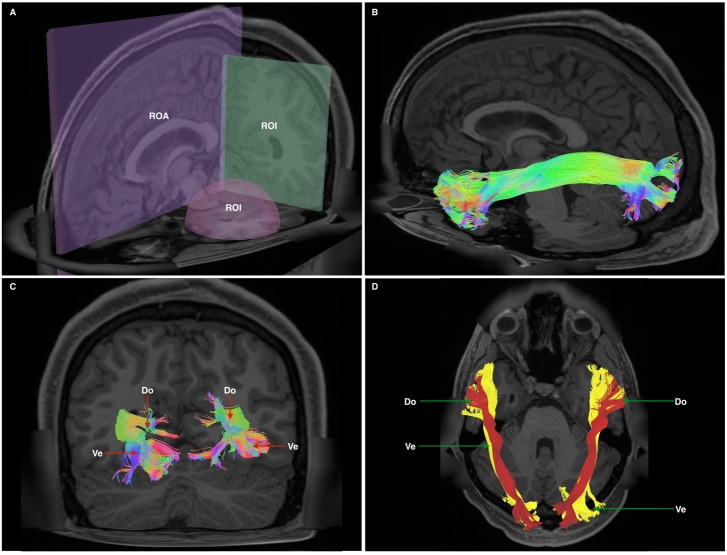
**(A)** Cutaway view showing all three radiological planes (axial, sagittal, and coronal) with ROIs and ROAs used. Spherical ROI is placed within the anterior temporal lobe white matter, whilst the rectangular ROI is placed in a coronal plane at the approximate position of the temporo-occipital junction. The ROA was placed in the mid-sagittal plane to exclude any fibers crossing the midline. **(B)** Generated whole, undivided ILF bundle after manual removal of spurious fibers belonging to other white matter systems and prior to separation. Color assignment is directional. **(C)** A posterior-coronal view demonstrating the posterior terminations of both left and right ILF bundles. Visible clearly is the bifurcated arrangement of each whole-ILF bundle which were subsequently used as geographical landmarks to separate the ILF further. Do, dorsal; Ve, ventral. **(D)** A superior-axial view demonstrating left and right ILF bundles following separation of the ILF into its respective subdivisions. The sub-fascicles have been individually colored. Do, dorsal (red); Ve, ventral (yellow).

Unlike our previous studies, and in an effort to replicate published methodology (Catani et al., 2003; Latini et al., 2017), we chose a two region of interest (ROI) approach rather than an atlas-based seeding approach. This method was chosen to minimize *a priori* selection of feed-backward or feed-forward tracts. A spherical ROI was positioned within the white matter of the anterior temporal lobe. A second, rectangular ROI was placed in the coronal plane at the temporo-occipital junction. To avoid commissural fibers and false continuities, a rectangular region of avoidance was placed across the sagittal hemispheric midline. Once 100,000 fiber tracts were generated, we manually removed fibers passing through the ventral external capsule [i.e., inferior fronto-occipital (IFOF), uncinate (UF) fasciculi] and fibers resembling the arcuate fasciculus (AF), superior longitudinal fasciculus (SLF) and middle longitudinal fasciculus (MdLF) (**Figure 1A**).

### Quantitative Connectivity Analysis and Connectogram Creation

We used a quantitative method to define cortical terminations. Each sub-fascicle was merged with its hemispheric counterpart (i.e., left and right). The ‘connectivity matrix’ function in DSI studio was used to generate matrices representing the number of fibers terminating within regions of a specially modified, per-subject aligned version of the Automated Anatomical Labeling (AAL) atlas. Three connectivity matrices were generated per subject, to give a total of 90 connectivity matrices corresponding to *whole ILF* (i.e., undivided), *dorsal ILF and ventral ILF* (see section “Results”) over the 30 individual subjects. These matrices were collated in Matlab and the number of fibers corresponding to each respective connection were divided by the total number of fibers for each fascicle, per subject. Values were then scaled over the range of 30 subjects to give a connection index (CI) between 0 and 100, with 0 representing no connectivity, and 100 representing strongest relative connectivity to a particular atlas region. Index values were then used to generate connectograms for each sub-fascicle and for whole ILF bundles, using CIRCOS^1^. Connectograms provide a unique method of visualizing network topology by demonstrating weighted strength of connectivity between brain regions.

### Volumetry and Lateralization

We calculated the number of voxels occupied by each fiber trajectory (streamlines) and the subsequent volume (in milliliters) of each gross ILF bundle and their manually separated subfascicles. The volumes of left and right merged ILFs and sub-fascicles across 30-subjects were each subjected to an independent samples *T*-test in SPSS (IBM Corporation, Armonk, NY, United States) to calculate significance of mean hemispheric volumetry over the 30 subjects. We also calculated the lateralization index (LI) using the formula [(volume left - volume right)÷(volume left + volume right)] × 2] (Catani et al., 2007).

### White Matter Dissection

Three human hemispheres were prepared for dissection. First, they were fixed in a 10% formalin solution for 2 months. After fixation, the arachnoid and superficial vessels were removed. The brains were subsequently frozen at -16°C for 2 weeks, as per the Klingler method (Ludwig and Klingler, 1956). Dissection commenced 24 h after the specimens were thawed and proceeded in a step-wise, superficial-to-deep process, at the lateral and inferior surface of the temporal lobe. Dissection was achieved using wooden spatulas to remove successive layers of gray matter followed by white matter. We utilized post-mortem white matter dissection only to study the ILFs’ position amongst other large association and projection white matter tracts, rather than for connectivity reasons.

## Results

### Morphology and Subdivision of ILF Bundles

**FIGURE 2:**
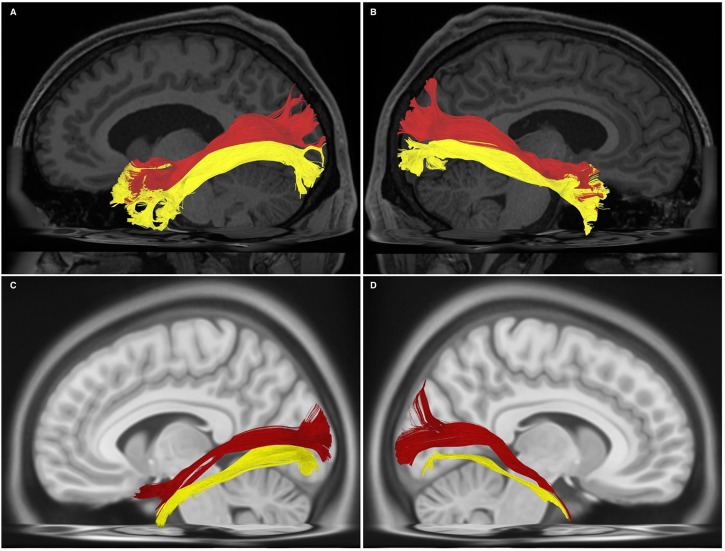
**(A)** Left-hemispheric, sagittal-view representation of a subdivided ILF in a single subject from the subject-specific analysis. DL, dorsal (red); VL, ventral (yellow). **(B)** Right-hemispheric, sagittal-view representation of a subdivided ILF in a single subject from the subject-specific analysis. DL, dorsal (red); VL, ventral (yellow). **(C)** Left-hemispheric, sagittal-view representation of a subdivided ILF in the HCP 1021 atlas. DL, dorsal (red); VL, ventral (yellow). **(D)** Right-hemispheric, sagittal-view representation of a subdivided ILF in the HCP 1021 atlas. DL, dorsolateral (red); VL, ventral (yellow).

Bundles resembling the ILF were found in 60/60 hemispheres (**Figure 1B**) in our subject-specific tractographic analysis and bilaterally within the 1021-subject template. Upon generating and extracting whole ILF bundles we observed a distinct posterior termination pattern across the individual subjects and in the template, which we used to further divide the ILFs (**Figure 1C**). Though the whole ILF bundles demonstrated a distinct posterior termination morphology, their anterior aspects did not. Upon morphological assessment, it was clear that the ILF followed a distinctive ‘dorsal-ventral’ termination pattern. We thus chose to manually separate these components, terming them *‘dorsal’ and ‘ventral,’* in addition to assessing the whole, unseparated bundle (**Figure 1D**). Both dorsal and ventral bundles were found in 60/60 of individual subjects’ hemispheres. Left and right ILFs were successfully replicated on the 1021-subject atlas and assumed identical morphology and subdivisions compared to those from the subject-specific study (**Figures 2A–D**).

### Quantitative ILF Connectivity

#### Dorsal ILF Sub-Fascicle (**Table 1**)

**Table 1:** Table of connectivity indices for left (blue) and right (green) dorsal ILF sub-fascicles.

Left	Tinf_L	TMd_L	TSp_L	Fu_L	Right	Tinf_R	TMd_R	TSp_R	Fu_R
Omd_L	0.00	7.90	26.08	0.00	Omd_R	0.00	0.00	0.00	0.00
Osp_L	9.10	41.13	100.00	6.18	Osp_R	7.77	12.59	11.47	8.54
Cal_L	0.00	0.00	7.01	0.00	Cal_R	0.00	0.00	0.00	0.00
Cun_L	0.00	5.74	33.14	0.00	Cun_R	5.41	11.83	25.99	0.00

*Darker color represents stronger index*.

**FIGURE 3:**
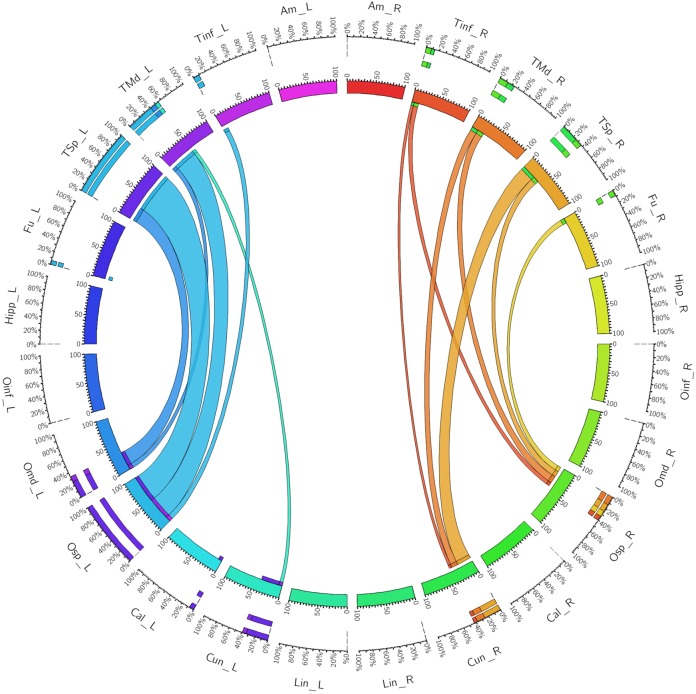
A connectogram representing bilateral connectivity patterns of the dorsal ILF subfascicle. Atlas regions are listed around the circumference of the connectogram. TInf, inferior temporal gyrus; TMd, middle temporal gyrus; TSp, superior temporal gyrus; Fu, fusiform gyrus; OInf, inferior occipital gyrus; OMd, middle occipital gyrus; OSp, superior occipital gyrus; Cal, calcarine gyrus; Cun, cuneus; Lin, lingual gyrus. Left and Right hemispheric connections are demonstrated with a suffix _L or _R.

The left dorsal ILF bundle demonstrated strongest connectivity patterns between the superior occipital to superior temporal gyri (CI: 100.00) and middle temporal gyri (CI: 41.13). All other connections had a CI <40. Right sided dorsal ILF connectivity was comparatively weaker versus the left: maximum connectivity was found between the cuneus and superior temporal gyrus (CI: 25.99), with all other connections demonstrating CI <25. Left dorsal ILF connectivity was spread over nine occipito-temporal connections, while the right was spread over 7. The average CI for the left dorsal ILF was 14.77 (*SD* = 26.13), while it was 10.45 (*SD* = 15.83) for the right (Not significant, *T* = 0.61, *p* = 0.547). Median CI for the left dorsal ILF was 23.10 and was 5.90 on the right (**Figure 3**).

#### Ventral ILF Sub-Fascicles (**Table 2**)

**Table 2:** Table of connectivity indices for left (blue) and right (green) ventral ILF sub-fascicles.

Left	Tinf_L	TMd_L	TSp_L	Fu_L	Hipp_L	Am_L	Right	Tinf_R	TMd_R	TSp_R	Fu_R	Hipp_R	Am_R
Fu_L	13.88	15.13	3.30	0.00	0.00	0.00	Fu_R	10.00	11.66	5.64	0.00	0.00	0.00
Oinf_L	37.83	40.89	35.62	3.92	0.00	0.00	Oinf_R	28.21	25.09	27.86	7.59	3.01	0.00
Omd_L	24.72	32.87	46.50	0.00	0.00	0.00	Omd_R	0.00	3.43	7.95	0.00	0.00	0.00
Osp_L	3.28	5.98	0.00	0.00	0.00	0.00	Osp_R	0.00	0.00	0.00	0.00	0.00	0.00
Cal_L	55.24	63.17	58.29	3.58	0.00	0.00	Cal_R	12.41	16.06	12.14	2.90	0.00	3.71
Cun_L	6.83	6.35	0.00	0.00	0.00	0.00	Cun_R	0.95	0.00	0.00	0.00	0.00	0.61
Lin_L	64.86	100.00	74.63	7.02	0.00	0.00	Lin_R	48.77	88.66	41.34	13.48	0.00	3.65

*Darker color represents stronger index.*

**FIGURE 4:**
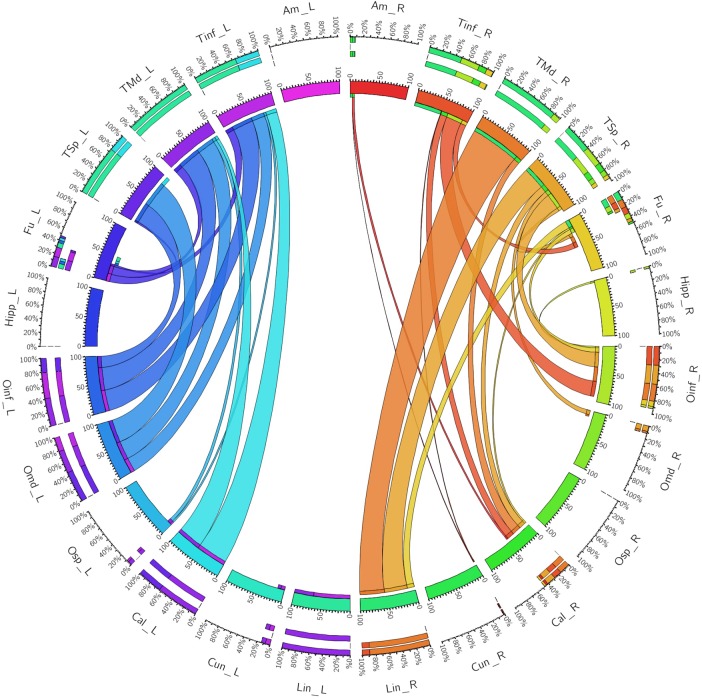
A connectogram representing bilateral connectivity patterns of the ventral ILF subfascicle. Atlas regions are listed around the circumference of the connectogram. TInf, inferior temporal gyrus; TMd, middle temporal gyrus; TSp, superior temporal gyrus; Fu, fusiform gyrus; OInf, inferior occipital gyrus; OMd, middle occipital gyrus; OSp, superior occipital gyrus; Cal, calcarine gyrus; Cun, cuneus; Lin, lingual gyrus. Left and Right hemispheric connections are demonstrated with a suffix _L or _R.

The left ventral ILF demonstrated strongest connectivity between the lingual gyrus and middle temporal gyrus (CI: 100.00), followed by lingual to superior temporal gyrus (CI: 74.63), and lingual to inferior temporal gyrus (CI: 64.86). Left ventral ILF also demonstrated strong connectivity between the calcarine area to middle temporal (CI: 63.17), superior temporal (CI: 58.29) and inferior temporal (CI: 55.24) gyri. All other left-sided ventral connections had CI’s under 50. The right ventral ILF demonstrated relatively weaker connectivity than the left, but with similar patterns: Lingual gyrus to middle temporal (CI: 88.66), inferior temporal (CI: 48.77) and superior temporal (CI: 41.34) gyri. All other connections had CI less than 40. Left ventral ILF demonstrated 22 distinct connections, whilst the right demonstrated 21. The average CI for the left ventral ILF was 16.76 (*SD* = 25.80) and 8.93 (*SD* = 17.10) for the right (not significant, *T* = 1.64, *p* = 0.053). Median CI for the left ventral ILF was 3.29. It was 0.78 for the right (**Figure 4**).

#### Whole ILF Bundles (**Table 3**)

**Table 3:** Table of connectivity indices for left (blue) and right (green) whole ILFs.

Left	Tinf_L	TMd_L	TSp_L	Fu_L	Am_L	Right	Tinf_R	TMd_R	TSp_R	Fu_R	Am_R
Fu_L	8.35	10.48	0.00	0.00	0.00	Fu_R	0.00	11.12	6.89	0.00	0.00
Oinf_L	22.02	29.09	30.52	0.00	0.00	Oinf_R	11.35	18.75	26.82	5.52	0.00
Omd_L	28.62	40.63	59.24	0.00	0.00	Omd_R	0.00	0.00	9.74	0.00	0.00
Osp_L	10.88	32.31	77.81	0.00	0.00	Osp_R	5.67	8.39	10.28	0.00	0.00
Cal_L	55.53	69.94	63.18	6.59	0.00	Cal_R	11.46	15.90	14.45	0.00	5.29
Cun_L	7.63	9.40	24.18	0.00	0.00	Cun_R	0.00	8.26	21.75	0.00	0.00
Lin_L	55.38	100.00	77.67	6.52	0.00	Lin_R	41.05	77.54	40.70	14.26	5.90

*Darker color represents stronger index. (NB, Left and Right hemispheric connections are demonstrated with a suffix _L or _R). TInf, inferior temporal gyrus; TMd, middle temporal gyrus; TSp, superior temporal gyrus; Fu, fusiform gyrus; OInf, inferior occipital gyrus; OMd, middle occipital gyrus; OSp, superior occipital gyrus; Cal, calcarine gyrus; Cun, cuneus; Lin, lingual gyrus; Am – amygdala; Hipp, hippocampus.*

**FIGURE 5:**
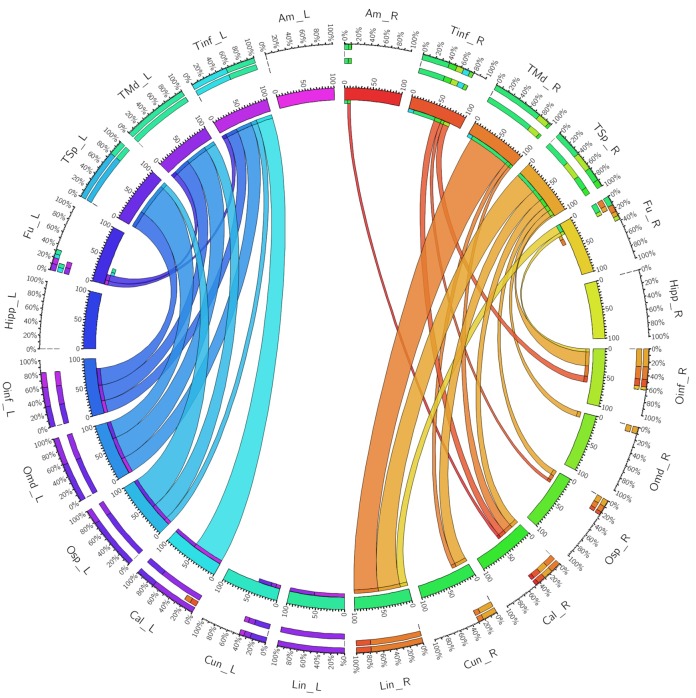
A connectogram representing bilateral connectivity patterns of the whole, unseparated ILF. Atlas regions are listed around the circumference of the connectogram. TInf, inferior temporal gyrus; TMd, middle temporal gyrus; TSp, superior temporal gyrus; Fu, fusiform gyrus; OInf, inferior occipital gyrus; OMd, middle occipital gyrus; OSp, superior occipital gyrus; Cal, calcarine gyrus; Cun, cuneus; Lin, lingual gyrus. Left and Right hemispheric connections are demonstrated with a suffix _L or _R.

**FIGURE 6:**
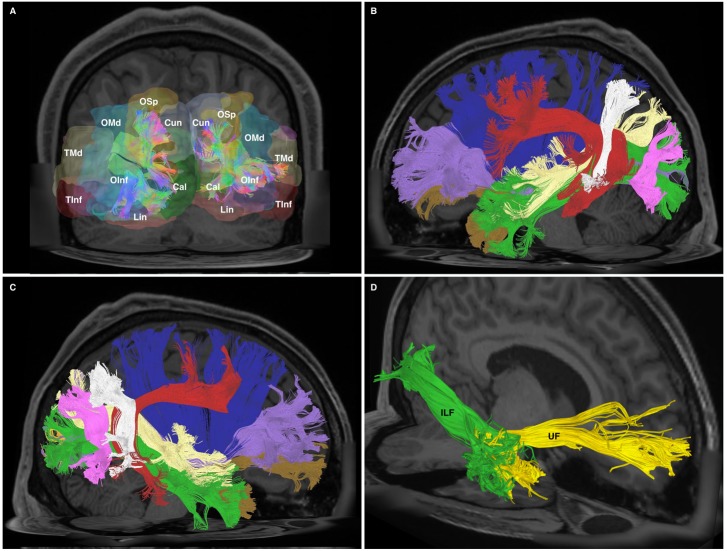
**(A)** Posterior-coronal view demonstrating occipito-temporal terminations of all divisions of the ILF. OSp, superior occipital gyrus; OMd, middle occipital gyrus; OInf, inferior occipital gyrus; Lin, lingual gyrus; Cun, cuneus; Cal, calcarine gyrus; TMd, middle temporal gyrus; TInf, inferior temporal gyrus. **(B)** Left-hemispheric sagittal-view representation of relational white matter tracts. Tracts are colored separately. IFOF, purple; Claustrum, blue; AF, red; PAT, white; MdLF, yellow; ILF, green; UF, brown; VOF, pink. **(C)** Right-hemispheric sagittal-view representation of relational white matter tracts. Tracts are colored separately. IFOF, purple; Claustrum, blue; AF, red; PAT, white; MdLF, yellow; ILF, green; UF, brown; VOF, pink. **(D)** An oblique-frontal view of the ILF (green) and UF (yellow) with focus of anterior temporal terminations. Occupying the medial white matter of the anterior temporal lobe are UF ventral terminations. The lateral white matter of the anterior temporal lobe consists of fibers which are part of the ILF.

When taken as whole, merged bundles, both left and right ILFs each demonstrated 22 patterns of occipito-temporal connectivity, respectively. The left ILF, however, demonstrated stronger connectivity indices versus the right. On the left, strongest connectivity was demonstrated between lingual to middle temporal gyrus (CI: 100.00), superior occipital to superior temporal gyrus (CI: 77.81), lingual to superior temporal gyrus (CI: 77.67), calcarine to middle temporal gyrus (CI: 69.94) and calcarine to superior temporal gyri (CI: 63.18). All other connections had CI <60. The strongest connections on the right side were between lingual and middle (CI: 77.54), inferior (CI: 41.05) and superior (CI: 40.70) temporal gyri. All other connections had a CI <40. The average CI for the whole ILF was 29.50 (*SD* = 29.29) on the left, while it was 10.60 (*SD* = 15.83) for the right (significant, *T* = 3.07, *p* = 0.004). Median CI for the left ILF was 23.10 and was 5.90 for the right (**Figures 5**, **6A**).

### Volumetry and Lateralization

Whole, merged ILF bundles had a mean volume of 19.1 ml (*SD* = 6.0), while right sided whole ILFs had a mean volume of 14.1 ml (*SD* = 5.4). This 5 ml difference was significant (*T* = 3.38, *p* = 0.001). For separated sub-fascicles, mean left dorsal ILF volume was significantly larger than right; at 9.8 ml vs. 6.9 ml, respectively (*T* = 3.50, *p* = 0.001). For the ventral ILF, mean left sided volume was 14.7 ml (*SD* = 5.5). For the right ventral ILF it was 10.9 ml (*SD* = 5.6). This hemispheric difference was significant (*T* = 2.64, *p* = 0.005). For whole ILF bundles, LI was 0.31 demonstrating leftward asymmetry. Both dorsal and ventral ILFs’ had a mean LI of 0.3, respectively. Using the HCP 1021 atlas, the whole ILF was 17.7 ml on the left and 11.0 ml on the right. When divided, the left dorsal ILF was 8.8 ml and the left ventral ILF was 11.1 ml. The right dorsal ILF was 2.4 ml, whilst the right ventral ILF was 9.4 ml.

### White Matter Tract Relations as Revealed by Tractography

**FIGURE 7:**
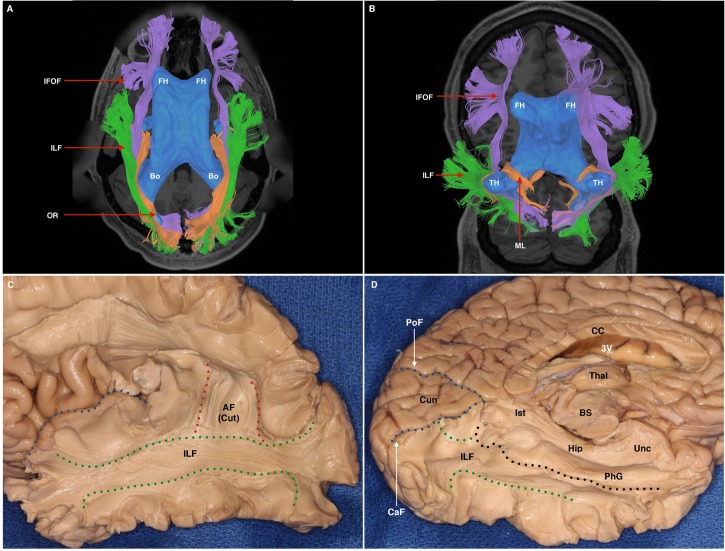
**(A)** Superior axial-view representation of tracts contributing to the sagittal stratum and their relation to the ventricular system. The ventricles are represented in blue as a 3-dimensional structure superimposed upon the axial T1 image. AH, anterior horn of the lateral ventricle; Bo, body of the lateral ventricle. IFOF, purple; ILF, green; OR, orange. **(B)** Inferior para-axial representation of tracts contributing to the sagittal stratum and their relation to the ventricular system. The ventricles are represented in blue as a 3-dimensional structure superimposed upon the axial T1 image. AH, anterior horn of the lateral ventricle; TH, temporal horn. IFOF, purple; ILF, green; ML, Meyer’s loop, passing over the superior surface of the temporal horn. **(C)** White matter dissection within a post-mortem specimen. Lateral-view. A portion of the Sylvian fissure is visible (blue broken line). At the temporal and parietal lobe, overlying cortex, U-fibers have been removed to expose the white matter of the ILF (demarcated by green broken lines). Some fibers of the dorsal portion of the AF, which overlies the ILF, are still visible (labeled AF Cut). **(D)** White matter dissection within a post-mortem specimen. View of infero-medial hemispheric surface. The corpus callosum (CC) and 3rd ventricle (3V) have been transected to achieve this view. The medial surface of the left thalamus is visible, which is situated superior to the superior brainstem (BS), which has also been transected. The cuneus (Cun) is a triangular-shaped lobe when viewed from the medial hemispheric surface. It is bounded superiorly by the parieto occipital fissure (PoF) and inferiorly by the calcarine fissure (CaF). Superficial structures have been removed to expose the ILF (between broken green (inferior) and black (superior) lines. Superior to the broken black line is the hippocampus (Hip), the calcar avis (CA). Dorsal to these structures are the isthmus (Ist), parahippocampal gyrus (PhG), and the uncus (Unc).

At its anterior aspect, The ILF shares terminations within the temporal pole with the ventral UF. It then travels posteriorly, lateral to the temporal horn. At its longitudinal-middle segment, temporal connections of the AF overlie it. Originating from the superior temporal gyri, the MdLF passes deep to the posterior limit of the Sylvian fissure and the AF, traveling obliquely to the dorsal occipital and superior parietal area, remaining dorsal to the ILF at all stages. At the temporo-occipital junction, the ILF travels posteriorly, joining the sagittal stratum inferiorly and remaining ventro-lateral to the IFOF. As such, the IFOF lies between the ILF and optic radiations, all of which traverse antero-posteriorly via the sagittal stratum to occipital terminations. As it passes over the basal temporal areas, i.e., the fusiform gyrus, the ILF is overlaid by the vertical occipital fasciculus (VOF), a short vertically oriented white matter tract originating from the basal occipito-temporal areas and connecting with the dorsolateral occipital gyri. At its posterior and occipital aspect, the ILF shares termination areas with the IFOF, MdLF, VOF and the optic radiations (**Figures 6B,C**, **7A,B**).

### White Matter Relations as Revealed by Dissection

From the lateral surface, the dissection started at the intersecting region of the parieto-occipito-temporal junctions, removing the U-fibers overlying the supramarginal and angular gyri. The vertical part of the SLF, also known as the parietal aslant tract, was exposed and removed to further expose the temporal AF, which was subsequently removed to expose underlying ILF fibers. ILF fibers were exposed along their antero-posterior course to the occipital pole. Toward the occipital pole, ascending fibers belonging to the VOF, were removed. From an inferior aspect U-fibers from the fusiform gyrus were gently removed to expose longitudinal ILF fibers. Lateral and medial occipito-temporal sulci were opened, as well as the lingual and parahippocampal gyri. The ILF represented the most lateral and ventral tract among other fascicles within the sagittal stratum. Its bifurcation in the sagittal plane led to identification of two components: ventral and dorsal. Dissection results reinforced the tractographic results: The ILF lays ventro-laterally to the posterior sagittal stratum fibers, all coursing in parallel to terminate within the occipital lobe. The ILF ran lateral to the temporal horn and its floor. At the temporal pole, the ILF ended lateral to the UF. At the occipital pole, it was flattened between the VOF laterally, and the IFOF medially (**Figures 7C,D**).

## Discussion

### ILF Morphology, Subdivisions and Relations

We have demonstrated that GQI-tractography can reproduce bilateral ILF bundles according to previous descriptions. Our results confirm the existence of a robust, temporo-occipital fascicle originating from medial and lateral anterior temporal areas and terminating throughout the occipital lobe. We did not find any evidence of the ‘indirect’ U-fiber component of the ILF (Tusa and Ungerleider, 1985; Catani et al., 2003). Further, we were able to divide the whole bundles into smaller sub-fascicles for individual study. We used the clear posterior bifurcation of the ILF into ventral and dorsal terminations in the temporo-occipital cortex. Though the ventral sub-fascicle itself demonstrated a bi-pronged ‘lateral-medial’ termination pattern, we elected to simplify our approach using dorsal and ventral divisions for further analysis. For both left and right hemispheric ILFs, posterior arrangement was consistent over 30 subjects and within the HCP 1021 atlas. Anterior ILF termination pattern was relatively more variable and did not demonstrate consistent inter-subject morphology, permitting subdivision. In terms of morphology and subdivision, our results are generally consistent with both older (Catani et al., 2002, 2003; Fernández-Miranda et al., 2008) and more recent (Latini, 2015; Latini et al., 2017) studies, all of which demonstrate posterior terminations within both dorsal (lateral and medial occipital cortex) and ventral (i.e., lateral, temporo-occipital areas and medial, basal-occipital areas). Previous authors have elected to subdivide the ILF based upon connectivity profiles. For example, Catani et al. (2003), Latini (2015), and Latini et al. (2017) defined a ‘cuneate’ branch of the dorsal ILF. From our connectivity analysis, CI’s were relatively small for cuneate connections of the left and right dorsal, ventral and undivided ILFs, compared to other connections. Using connective rather than morphological subdivisions introduces complexity into a nomenclature system, as an arbitrary threshold must be chosen to define what CI qualifies a connection as a significant ‘subdivision’ of the ILF. Regarding relations, we confirm that the ILF is the most superficial and ventral white matter fasciculus of the temporal lobe. Though cortico-cortical U-fibers may lie superficial to the robust ILF and indirectly connect temporal with occipital areas via short connections, we have found no tractographic evidence to suggest their anatomical affiliation with the ILF system.

### Quantitative ILF Connectivity

We used a purely quantitative method of assessing the connectivity of the ILF and its sub-fasciculi. This represents a methodological evolution from our previous studies (Wang et al., 2013, 2016; Fernández-Miranda et al., 2015; Panesar et al., 2017), all of which used qualitative correlations of fiber end-points within atlas regions. Not only does our quantitative technique give an objective measure of cortical termination, it also allows a metric analysis of the strength of connectivity between cortical areas. The need for quantitative termination analysis methods in tractographic studies has been identified (Girard et al., 2014) and will ultimately permit mapping of the connectome (Behrens and Sporns, 2012). Recently published quantitative analysis methods include statistical analysis of tract termination patterns derived from whole brain probabilistic DTI tractograms (Hau et al., 2016, 2017). Our use of connectograms with deterministic tractography (Yeh et al., 2018) aids understanding of connectivity strength by visually demonstrating bilateral ILF temporo-occipital connectivity in a topographically correlated matrix.

From our connectivity analysis of individual sub-fasciculi, it was clear that both sub-fascicles, and the undivided ILF were leftward-lateralized in terms of connectivity. This is confirmed by an overall increased average connectivity strength on the left versus the right for individual sub-fascicles. Though the differences in connectivity between left and right ILF subcomponents were not statistically significant, we postulate that this arose due to a relatively small size of the subject set and large spread of CIs. Moreover, though there were some fibers traversing between the cuneus, lingual gyri, and the amygdala, these connections were negligible in relative CI (see also section “ILF Morphology, Subdivisions and Relations”). We therefore question whether the ‘Li-Am’ bundle as proposed by Latini (2015) or ‘cuneate’ bundle as proposed by Catani et al. (2003) should be attributed as unique subdivisions of the ILF, versus simple connections.

Our connectivity results demonstrate the novel finding of strong leftward connectivity of the ventral ILF between the calcarine area and all three temporal gyri. This is in contrast to existing views (Catani et al., 2003; Latini, 2015; Latini et al., 2017). Further, connections originating from the calcarine area had strong relative CIs for the ventral sub-fascicle, and also when the undivided ILF was analyzed as a whole. The functional implications of these connections are discussed in Section “ILF Volumetry.” Another novel finding is minimal or absent connectivity with the antero-medial temporal areas, which is again in contrast to previous postulations (Catani et al., 2003; Mills et al., 2013; Latini et al., 2017; Jennings et al., 2018). We postulate that this is because medial anterior-temporal white matter trajectories are occupied primarily by the uncinate fasciculus (**Figure 6D**). The ventral uncinate fasciculus descends via the temporal stem and occupies medial antero-temporal white matter trajectories (Panesar et al., 2017). As the ILF is the most superficial, ventral white matter tract, its connectivity is primarily to the lateral cortical aspects of the temporal lobe. As all of these studies used DTI, we attribute previous findings of ILF antero-medial temporal connectivity to inability of DTI to accurately discern close proximity fiber systems at a high resolution versus our GQI method.

### ILF Volumetry

Our volumetric results, demonstrating significant leftward asymmetry of the whole ILF, and its individual constituents are in concordance with earlier DTI-derived results (Wakana et al., 2007; Thiebaut de Schotten et al., 2011), however, it was in contrast to recent postulations by Latini et al. (2017), which demonstrated rightward volumetric asymmetry. Our results of leftward volumetric asymmetry can be attributed to overall increased leftward volume of both ILF subcomponents, which all demonstrate LIs >0. Our results are in concordance with our previous study into IFOF structure, which also demonstrated leftward lateralization of this ventral tract. The ILF and IFOF may therefore be both structurally and functionally related.

### Functional Postulations

Regarding function of individual subfascicles, the left dorsal ILF demonstrated strongest connectivity between the superior occipital gyrus and the two dorsal temporal gyri. The superior temporal gyrus has been implicated in emotional responses to visual facial stimuli (Domínguez-Borràs et al., 2009). The middle temporal gyrus has been implicated in both semantic access (Davey et al., 2015), distance judgment (Vandenberghe et al., 1996), and facial recognition (Haxby et al., 2000). The dorsal ILF is therefore strong candidate for a neural substrate subserving both semantic, spatial and facial recognition tasks. The left ventral sub-fascicle connected strongly with inferior and middle occipital gyri, cuneate, calcarine, and lingual areas, respectively. Both had extensive terminations within the three lateral temporal gyri. Right sided ventral ILFs demonstrated exclusive connectivity between lingual and the all three temporal gyri. The cuneus is implicated in extrastriate visual processing (Allison et al., 1994). The calcarine cortex contains V1, and the lingual gyrus has been implicated in both facial recognition (Haxby et al., 2000) on the right and binocular spatial orientation bilaterally (Lee et al., 2001). Our results therefore implicate bilateral ventral components of the ILF to be implicated in facial recognition and would explain lesion-induced prosopagnosia (Benson, 1974; Bauer, 1984; Michel et al., 1989; Takahashi et al., 1995; Grossi et al., 2014).

Within the ‘dorsal-ventral’ model of language functionality, prevailing opinion is of a leftward-lateralized ventral semantic system (Hickok and Poeppel, 2015). Our connective and volumetric results therefore support a proposal of the ILFs role within the ventral semantic-stream. Further, due to its rich occipito-temporal connectivity, our results also support a dominant ILF role as an integrative system between the visual cortices and the relevant lateral temporal gyri, as evidenced by lesional studies (Bonilha et al., 2017). Recent theories (Dehaene and Cohen, 2011; Zemmoura et al., 2015) have proposed the left-sided occipital visual word form area as a ‘functionally recycled’ neural substrate, originally evolved to subserve facial recognition. This theory is consistent with theories of ILF function, which implicate it in both semantic language and visual recognition roles. Our findings of ILF leftward-lateralization also reinforce this postulation.

### Technical Considerations and Limitations

Our method was intended to generate whole ILFs without *a priori* regarding either connectivity or subdivision. Subsequent separation of the bundles did indeed reveal a dorsal-ventral ILF arrangement in accordance with the literature. Nevertheless, based upon our connectivity results, nomenclature for subdivided sub-fasciculi should not be used to indicate connectivity patterns (see sections “ILF Morphology, Subdivisions and Relations,” “Quantitative ILF Connectivity”), but instead only morphological divisions of the ILF. This is exemplified by the right ventral ILF sub-fascicles, both of which demonstrate connectivity to both medial and lateral occipital cortices. Regarding connectivity overlaps between the divisions, whether these occur due to true segregation between sub-fasciculi or whether these are due to tractographic artifacts remains to be determined. In an effort to reconcile this issue, and also proposed by Latini et al. (2017), we emphasize that both connectivity and volumetric results for whole, merged ILFs take precedent over results for individual sub-tract analyses. Though connective segmentation of the ILF may seem appealing, especially using our CI metric, we opt not to use it to define specific ‘subdivisions’ of the ILF, as we cannot define the arbitrary threshold deeming a connection significant enough to be considered an independent sub-fasciculus rather than a common connection or artifact.

Our current high-resolution GQI based imaging technique gives ability to accurately track close-proximity and crossing fiber systems to termination points within cortical mantle. The combination of this technique with quantitative connectivity analyses is the basis for connectogram construction and provides our most detailed and objective study to date. Further, when the pitfalls of DTI imaging are taken into consideration, the combination of tractography with objective connectivity analyses offers a superior solution for *in vivo* research studies. Post-mortem fiber dissection has played a pivotal role in neuroanatomical studies and remains a valuable technique for comparison and validation of tractographic findings. Nonetheless, post-mortem fiber dissection provides only qualitative evaluation and is prone to human errors. It is becoming rapidly surpassed by tractographic methods that allow for quantifying neuroanatomic parameters such as connectivity, and within large subject-sets as we have demonstrated.

## Conclusion

We have successfully replicated a subdivided ILF bundle consistently over the range of 30 subjects. Though our results bear resemblance to previous postulations regarding the subdivision and gross connectivity of the ILF, we have offered a detailed picture of its bilateral connectivity patterns. The ILF is a connectively and volumetrically leftward-lateralized, ventral temporal bundle with rich occipito-temporal connectivity. Though tractography cannot give direct functional insight, our connectivity analysis taken in context with functional data pertaining to cortical functions offer an accurate and confirmatory insight into its postulated roles. We conclude by calling for increased adoption of GQI tractography and quantitative connectometry for anatomical studies.

## Ethics Statement

This study was carried out in accordance with the recommendations and approval of the Institutional Review Board at the University of Pittsburgh. All subjects gave written informed consent in accordance with the Declaration of Helsinki.

## Author Contributions

SP contributed to the main data collection, writing, image creation, and tabulation. F-CY designed the algorithm, calculated the connectivity indices, and created the connectogram. TJ contributed to the WM dissection and pictures and discussion. WH contributed to the discussion. JF-M was the PI of the lab and contributed the main ideas, to document editing, and finalization.

## Conflict of Interest Statement

The authors declare that the research was conducted in the absence of any commercial or financial relationships that could be construed as a potential conflict of interest.

## Footnotes

1 http://mkweb.bcgsc.ca/tableviewer/visualize/
